# OX26/CTX-conjugated PEGylated liposome as a dual-targeting gene delivery system for brain glioma

**DOI:** 10.1186/1476-4598-13-191

**Published:** 2014-08-13

**Authors:** Pei-jian Yue, Lei He, Shu-wei Qiu, Yi Li, Yi-ji Liao, Xiang-pen Li, Dan Xie, Ying Peng

**Affiliations:** Department of Neurology, Sun Yat-sen Memorial Hospital, Sun Yat-sen University, NO.107, Yan Jiang Xi Road of Guangzhou, Guangzhou, 510120 China; Key Laboratory of Malignant Tumor Gene Regulation and Target Therapy of Guangdong Higher Education Institutes, Sun Yat-sen University, Guangzhou, 510120 China; State Key Laboratory of Oncology in South China, Cancer Center, Sun Yat-Sen University, NO.651, Dongfeng Road East, Guangzhou, 510060 China

**Keywords:** Dual-targeting, Gene therapy, Chlorotoxin, Blood–brain barrier, Glioma

## Abstract

**Background:**

The successful gene delivery into the brain is a major challenge due to the presence of the blood–brain barrier (BBB). In order to transport plasmid DNA across the BBB and target the brain glioma, the PEGylated liposomes (PLs) modified with OX26 and chlorotoxin (CTX) were developed as a dual-targeting gene delivery system, and the therapeutic efficacy of OX26/CTX-PL/pC27 against glioma was evaluated using *in vitro* and *in vivo* experimental models.

**Methods:**

The PEGylated liposome complexes were prepared by the reverse phase evaporation method, and their physicochemical properties were examined. The transfection efficiency, intracellular distribution, *in vitro* effects of OX26/CTX-PL/pC27 were determined on C6, F98 and HEK293T cell lines. The dual-targeting therapeutic efficacy of OX26/CTX-PL/pC27 against glioma were assessed using the BMVECs/C6 cells co-culture model and the rat orthotopic glioma model.

**Results:**

The OX26/CTX-PL/pDNA complexes exhibited a subglobose shape, and possessed notably low toxicities to HEK293T and C6 cells post 4 h incubation. In the *in vitro* transfection experiment, gene expressions of hTERTC27 from C6 and F98 cells were significantly improved by OX26 and CTX modification. Our *in vitro* results also showed that OX26 endowed the PLs with the transport ability across the BBB. Using the BMVECs/C6 cells co-culture model, the viability of C6 cells was decreased to 46.0% after OX26/CTX-PL/pC27 transfection. The OX26/CTX-PL/pC27 complexes exhibited enhanced therapeutic effects on C6 cells. Moreover, the dual-targeting therapeutic effects were further conformed with diminished tumor volumes (18.81 ± 6.15 mm^3^) and extended median survival time (46 days) in C6 glioma-bearing rats. Immunohistochemical analysis revealed the therapeutic effects derived from enhanced hTERTC27 expression in the tumor site.

**Conclusions:**

The PEGylated liposomes modified with OX26 and CTX are able to significantly promote cell transfection, increase the transport of plasmid DNA across the BBB and afterwards target the brain glioma cells *in vitro* and *in vivo*, exhibit the most significant therapeutic efficacy. The ligand OX26 plays a critical role in transporting the lipoplexes across the BBB, and CTX acts as a major role in targeting brain glioma cells. The results would encourage further developments for non-invasive targeting therapy of brain gliomas by intravenous injection.

**Electronic supplementary material:**

The online version of this article (doi:10.1186/1476-4598-13-191) contains supplementary material, which is available to authorized users.

## Background

Gliomas are the most frequent primary intracranial tumors in adults, and the yearly incidence is six cases per 100 000 [1]. The high-grade glioma, glioblastoma multiforme (GBM), almost invariably infiltrates the surrounding normal brain tissue, which makes it impossible for a complete surgical tumor removal, and is typically associated with rapid progression and a fatal outcome [2]. Another obstacle for the treatment of GBM is the presence of the blood–brain barrier (BBB), which prevents nearly all large-molecule drugs and more than 98% of small-molecule pharmaceuticals entering the brain tissue [3]. Over the past 3 decades, the standard treatment has evolved to include maximal safe surgical resection, radiation therapy and temozolomide chemotherapy, while the median survival of GBM patients only achieves 14.6 months. GBM remains to be lethal for most of the patients [4, 5].

Alternatively, gene therapy may offer a promising cure for various diseases including GBM. To obtain a better therapeutic effect, a gene delivery system is urgent for clinic employ to transport genes across the BBB and then target the brain glioma.

Liposomes, as an effective gene vehicle, are rapidly eliminated from the blood circulation by the mononuclear phagocyte system (MPS) after intravenous administration [6–8]. By conjugating polyethyleneglycol (PEG) to the surface of liposomes, the PEG outer shell can reduce protein opsonization and subsequent phagocytosis by the MPS, resulting in an increased circulation time [9]. This kind of sterically stable, long-circulating liposomes have led to a new era in the liposome drug delivery [10–12]. By coupling with targeting antibodies, peptides, and small molecules, such as OX26 [13], transferring (Tf) [14], angiopep-2 [15] and folate [16], the pegylated liposomes can actively target the tumor sites. Such a targeting gene delivery system is characterized with a high stability of the encapsulated DNA under physiological conditions and a prolonged circulation half life *in vivo*, and finally exerts greater antitumor activities, compared with the conventional liposomes loaded with therapeutic genes [8, 17, 18].

The transferrin receptor (TfR) is abundant on the brain capillary endothelium, one component of the BBB, and therefore the TfR monoclonal antibody (mAb) of rats, OX26, is able to bind to an extracellular domain of TfR and transport across the BBB via the endogenous transferrin transport system [17, 19]. And since the binding domain of OX26 is distinct from the transferrin binding site, it does not interfere with Tf binding under physiological conditions [20]. Thus, the OX26 is a very efficient targeting antibody candidate in brain drug delivery [21]. However, the TfRs express not only on the BBB and tumor cells, but also on neuronal plasma membrane as well as other organs, such as liver and spleen. As a result, the expression of exogenous plasmid DNA for this strategy reaches throughout the central nervous system, leading to a lack of the specific targeting to the tumor tissue [13, 17, 22].

Chlorotoxin (CTX), originally isolated from Leiurus quinquestriatus venom, is a 36-amino acid peptide tightly folded via four disulfide bridges. Several researches have indicated that CTX specifically binds to gliomas and tumors of neuroectodermal origin rather than non-neoplastic cells or normal brain [23, 24], and this specific binding to cancer cells is facilitated by matrix metalloproteinase-2 (MMP-2) [25]. These characteristics make this molecule an attractive targeting candidate for the diagnosis and treatment of tumors [26, 27].

hTERTC27, a 27 kDa C-terminal polypeptide of human telomerase reverse transcriptase (hTERT), is capable of inducing telomere dysfunctions, anaphase chromosome end-to-end fusions in hTERT-positive HeLa cells, and inhibits HeLa cell growth and tumorigenicity in nude mice xenografts [28]. Especially, hTERTC27 takes effect without perturbing the endogenous telomerase activity, thereby minimizing the potential side effects on telomerase positive reproductive cells and proliferative cells of renewal tissues in anti-telomerase therapies [29, 30].

In the present study, we developed a dual-targeting system, plasmid IRES2-EGFP/hTERTC27 (pC27)-loaded PEGylated liposomes (PLs) modified with OX26 and CTX (OX26/CTX-PL/pC27) as a platform for the targeting delivery of pC27 to glioma. This system is expected to transport pC27 across the BBB and then target brain glioma mediated by OX26 and CTX, respectively. The objective of this study is to determine the therapeutic efficacy of OX26/CTX-PL/pC27 in the treatment of glioma.

## Results and discussion

Successful gene delivery into the brain is a major challenge due to the presence of the BBB. Over the last decade, a number of ligands, such as OX26, folate and angiopep-2, have been verified capable of specifically binding to surface receptors on the target sites, resulting in increased selectivity [31, 32]. Accordingly, there have been great developments concerning the brain drug delivery systems [14, 33, 34]. However, most of them are developed for delivering daunorubicin, paclitaxel, or other widely used chemotherapeutics in glioma therapy. In the present study, we employed the PEGylated liposomes as the delivery vector of plasmid DNA and constructed the dual-targeting gene delivery system.

**Figure 1:**
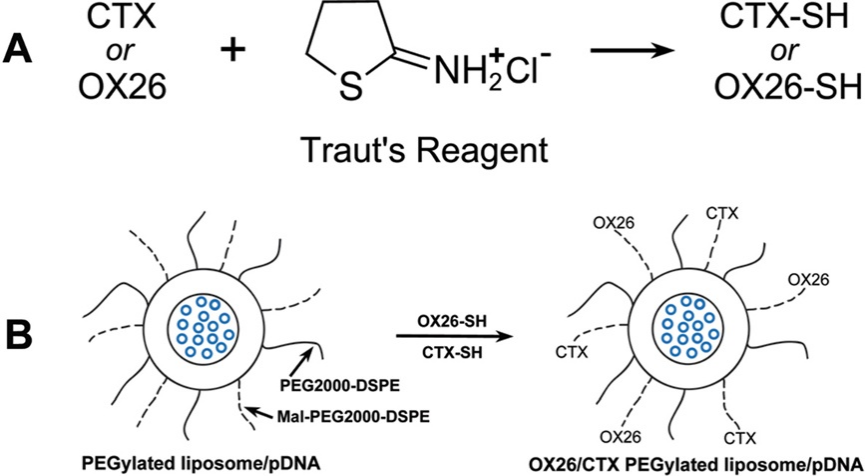
**The schematic diagram for the synthesis of targeting PL/pDNA complexes. (A)** The OX26 and CTX were thiolated by 2-iminothiolane (Traut’s reagent) at room temperature for 1 hour. **(B)** The outer maleimide groups of PL/pDNA react with the thiolated OX26 and CTX to form targeting PL/pDNA complexes.

OX26-PL/pDNA, OX26/CTX-PL/pDNA complexes were prepared through the outer maleimide groups of PLs specifically reacting with the thiol groups of OX26-SH and CTX-SH (Figure 1). To explore the effects of the ratios of DC-chol to pDNA on the particle size and zeta potential, we prepared PL/pDNA complexes with different weight ratios under the condition of fixing the amount of S100-PC and pDNA. As the weight ratio of DC-chol/pDNA reached 6:1, the particle size tended to be stable (Additional file 1: Figure S1).

The mean particle size of all PL/pDNA complexes was approximately 120 nm, with an appropriate polydispersity index (PDI, approximately 0.2). All of the charge values were positive, and this guaranteed high pDNA encapsulation efficiency by electrostatic attraction of opposite charges (Additional file 2: Table S1). The PL/pDNA complexes exhibited subglobose shape of moderate uniform particle size and there was no aggregation phenomenon of the particles. The particle size measured from the TEM images was in good agreement with that measured by the laser scattering method (Additional file 3: Figure S2). No significant change in the mean particle size of the liposome complexes was detectable after 1 week when kept in PBS at 4°C. In DMEM containing 10% FBS, the liposome complexes remained stable in terms of size for 8 h at 37°C (data not shown).

The size of particles plays a critical role in their clearance by the spleen. Since the interendothelial cell slit (IES) size of the spleen rarely exceeds 200 to 500 nm in width, particles must be either small or deformable enough to avoid the splenic filtration through the IES of venous sinuses. Therefore ideally, the size of particles should not exceed 200 nm [35, 36]. Besides, it has been reported that the nanoparticles with a mean diameter approximately 100 nm showed prolonged blood circulation and a relatively low rate of MPS uptake [37]. The mean diameter of the different PL/pDNA complexes in this study was approximately 120 nm, which satisfied the size requirements described above. The prolonged blood circulation attributed to make more nanoparticles reach the brain and achieved enhanced permeability and retention (EPR) effects.

Positively charged particles can fully condense and coat DNA, thus avoiding the degradation of DNases [38]. However, such particles tend to form small aggregates in the presence of the negatively charged serum proteins once i.v. administered and often exhibit a rapid blood clearance phase with a large dose accumulating in the lung and the liver. Grafting neutral PEG onto the surface of the cationic liposomes prevents aggregation and increases their stability due to the charge shielding [39].

The encapsulation efficiency of all lipoplexes was show in Additional file 4: Table S2. Compared with PL/pEGFP or PL/pC27, the encapsulation efficiency of OX26-PL/pDNA, OX26/CTX-PL/pDNA decreased to some extent. It may be a consequence of pDNA releasing from complexes during the reaction between OX26, CTX and PL/pDNA. The coupling efficiencies of OX26 in OX26-PL/pC27, OX26/CTX-PL/pC27 were 40.4 ± 5.1% and 35.4 ± 3.8%, respectively. The coupling efficiencies of CTX in CTX-PL/pC27, OX26/CTX-PL/pC27 were 44.9 ± 6.1% and 42.5 ± 5.5%, respectively. There was no obvious interaction between OX26 and CTX during the coupling reaction.

The cytotoxicity of the PL/pEGFP, OX26-PL/pEGFP and OX26/CTX-PL/pEGFP complex at a series of plasmid weights were evaluated with CCK-8 Kits in HEK293T and C6 cells (Additional file 5: Figure S3). Cell viability decreased as the weight of pEGFP in the complex increased. For HEK293T, cell viability of OX26/CTX-PL/pEGFP group was 94.05% ± 0.43% and 82.94% ± 0.41% at 0.1 μg and 1.6 μg pEGFP, respectively. For C6, cell viability was 93.67% ± 0.47% and 81.89% ± 0.44% at 0.1 μg and 1.6 μg pEGFP, respectively. The PL complexes showed notably low toxicity to the HEK293T and C6 cells after 4 h incubation, which might be attributed to the similarity of the liposomes to the cell membranes and the charge-shielding effects of PEG chains. No statistical differences in the cell viability were found among PL/pEGFP, OX26-PL/pEGFP and OX26/CTX-PL/pEGFP at the corresponding plasmid weights except OX26/CTX-PL/pEGFP at 1.6 μg pEGFP (p < 0.01 compared with that of PL/pEGFP and OX26-PL/pEGFP, respectively).

**Figure 2:**
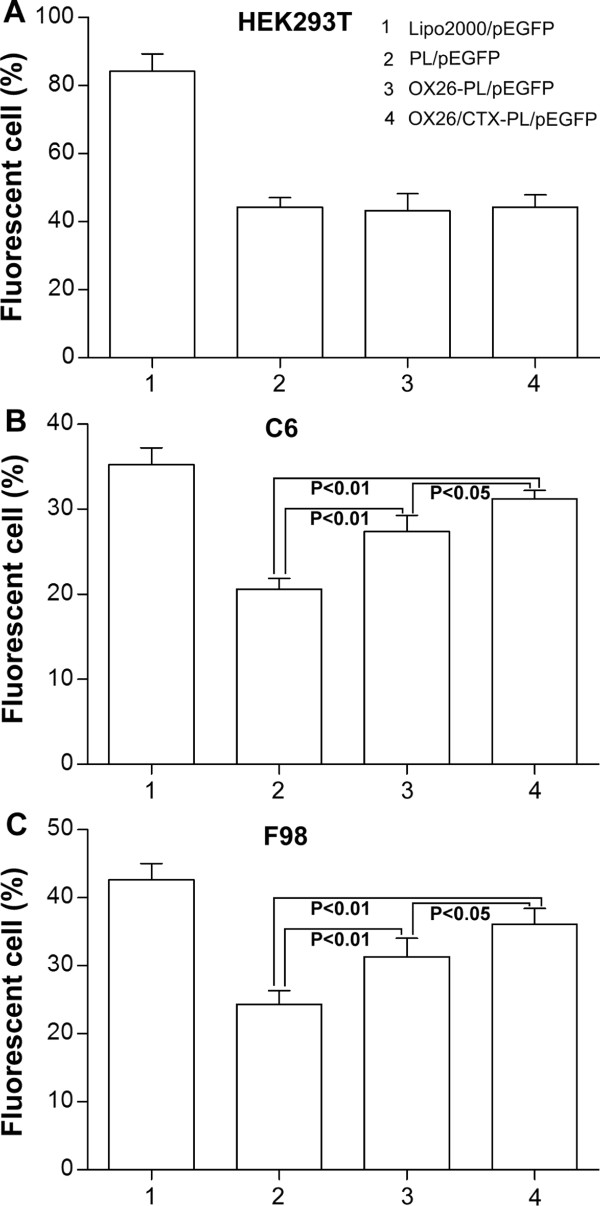
**Transfection efficiency of Lipo2000/pEGFP, PL/pEGFP, OX26-PL/pEGFP and OX26/CTX-PL/pEGFP complexes containing 1 μg pIRES2-EGFP by FACS analysis in (A) HEK293T cells, (B) C6 glioma cells, (C) F98 glioma cells.** Data are expressed as mean ± SD (n = 3).

The transfection efficiency of complexes was visualized by observation of EGFP positive cells using a fluorescence microscope. For HEK 293 T cells, no obvious differences of the fluorescent density were observed among PL/pEGFP, OX26-PL/pEGFP and OX26/CTX-PL/pEGFP containing 1 μg pEGFP. For C6 and F98 cells, the highest transfection efficiency was found in OX26/CTX-PL/pEGFP complex while the weakest fluorescent density was displayed in PL/pEGFP complex at 1 μg pEGFP (Additional file 6: Figure S4). The transfection efficiency was further quantified by the flow cytometry assay. When HEK293T cells were transfected with Lipo2000/pEGFP, PL/pEGFP, OX26-PL/pEGFP and OX26/CTX-PL/pEGFP complexes at 1 μg pEGFP, the average ratios of EGFP-positive cells were 84.2%, 44.3%, 43.2% and 44.3%, respectively (Figure 2A). However, the average ratios were 35.2%, 20.6%, 27.4% and 31.2% for C6 cells, and 42.6%, 24.3%, 31.3% and 36.1% for F98 cells, respectively (Figure 2B, 2C). No significance on transfection efficiency was shown in HEK 293 T cells (p > 0.05), while compared with that of PL/pEGFP for C6 and F98 cells, higher transfection efficiencies of OX26-PL/pEGFP and OX26/CTX-PL/pEGFP were indicated (p < 0.01) (Figure 2). This was probably attributed to the absence of the corresponding receptors of OX26 or CTX on HEK293T cells, and the recognition of OX26 to TfR, CTX to MMP-2 in C6 and F98 cells (both TfR and MMP-2 are over expressed in the rat glioma cells).

To quantify the transgene expression level, luciferase reporter assays were carried out. As the increase of the weight of pGL3-luc, enhanced luciferase activities were shown among Lipo2000/pGL3-luc, PL/pGL3-luc, OX26-PL/pGL3-luc and OX26/CTX-PL/pGL3-luc. For C6 and F98 cells, OX26-PL/pGL3-luc and OX26/CTX-PL/pGL3-luc complexes obtained obvious higher luciferase activities than the PL/pGL3-luc complexes among all the tested weights of pGL3-luc, and there was also obvious difference between OX26-PL/pGL3-luc and OX26/CTX-PL/pGL3-luc (Additional file 7: Figure S5B, C), indicating that the novel OX26/CTX-PL/pGL3-luc complexes increased the transgene expression due to its targeting ligands. No differences were found in luciferase activities among the three complexes above in HEK293T cells (p > 0.05) (Additional file 7: Figure S5A). Consistent with results from the flow cytometry assay, this further proved the possible targeting effect of OX26 and CTX.

Intracellular expression and distribution of hTERTC27 was observed by virtue of confocal microscope (Additional file 8: Figure S6). The expression of hTERTC27 presented in a time-dependent manner. With extension of culture time, positive cells and fluorescence intensity of gene expression increased. The hTERTC27 protein distributed throughout the C6 cells, though primarily within the cell nuclei.

**Figure 3:**
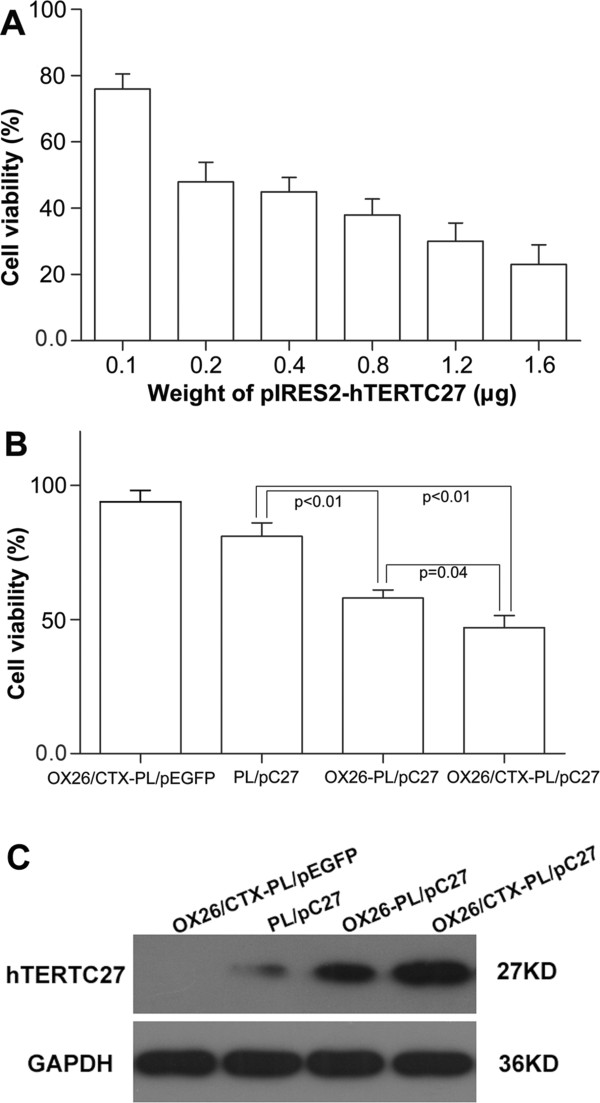
**The therapeutical effect of OX26/CTX-PL/pC27 complex and western blot analysis. (A)** The cell viability of the C6 glioma cells after transfection of OX26/CTX-PL/pC27 complex at a series of plasmid weights. **(B)** The viability of C6 glioma cells after transfection of OX26/CTX-PL/pEGFP, PL/pC27, OX26-PL/pC27 and OX26/CTX-PL/pC27 at 0.2 μg pDNA. **(C)** Western blot analysis of the expression of hTERTC27 in C6 glioma cells after transfections of OX26/CTX-PL/pEGFP, PL/pC27, OX26-PL/pC27 and OX26/CTX- PL/pC27complexes at 4 μg pDNA. OX26/CTX-PL/pEGFP was set as the negative control.

To explore the potential therapeutical effect of OX26/CTX-PL/pC27 complex, its cytotoxicity was initially evaluated at a series of plasmid concentrations in C6 glioma cells by CCK-8 assay. As shown in Figure 3A, reduction of cell viability was shown with the increase of weight of pIRES2-hTERTC27 in the complex. Figure 3B showed the viability of C6 glioma cells treated with PL/pC27, OX26-PL/pC27 and OX26/CTX-PL/pC27 at 0.2 μg pC27 (81% ± 5%, 58% ± 3% and 47% ± 4.5%, respectively). Obviously, OX26-PL/pC27 and OX26/CTX-PL/pC27 exhibited higher toxicity to C6 glioma cells than PL/pC27, and there was also obvious difference between OX26-PL/pC27 and OX26/CTX-PL/pC27 (p = 0.04). The results indicated that the strength of antitumor effects was OX26/CTX-PL/pC27 > OX26-PL/pC27 > PL/pC27. The results may be explained by the higher hTERTC27 protein level in the OX26/CTX-PL/pC27 and OX26-PL/pC27 treatment groups (Figure 3C). Therefore, we concluded that targeting ligands OX26 and CTX promoted cell transfection of lipoplexes, increased hTERTC27 protein expression, and enhanced the therapeutical effect on C6 cells.

**Figure 4:**
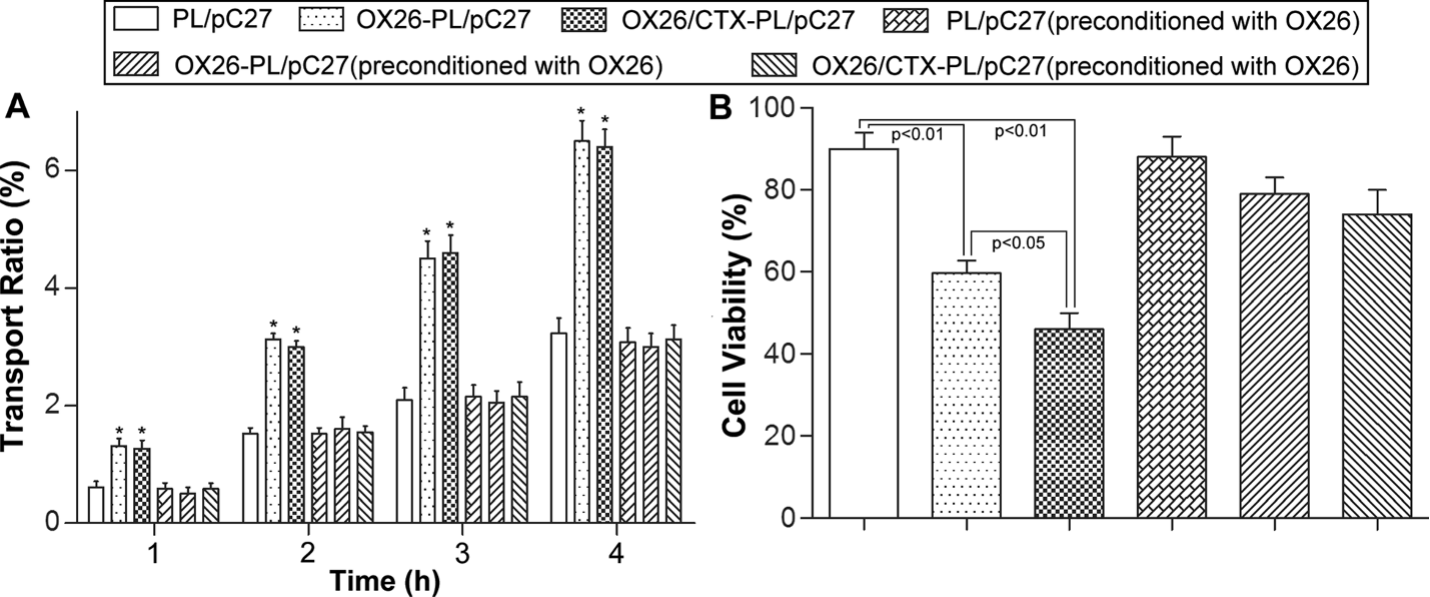
**Transport ratios across the BBB and dual-targeting effects** ***in vitro*** **. (A)** The transport ratios of pC27 across the BBB during 1 ~ 4 hour. **(B)** The cell viability of C6 cells after pC27 transported across the BBB. Data were presented as mean ± SD (n =3). *p < 0.01, vs. PL/pC27.

*In vitro* BBB model was established and confirmed by the permeation experiment and transendothelial electrical resistance (TEER) values (>250 Ωcm^2^). No obvious reduction of TEER values was observed during the experiment, indicating that transport of pC27 did not disrupt the BBB barrier. Figure 4A showed the transport ability of different lipoplexes across BBB model at the same concentration of pDNA. The transport ratios were 3.23% for PL/pC27, 6.50% for OX26-PL/pC27, 6.42% for OX26/CTX-PL/pC27, 3.08% for PL/pC27 preconditioned with OX26, 3.00% for OX26-PL/pC27 preconditioned with OX26, 3.13% for OX26/CTX-PL/pC27 preconditioned with OX26 at 4 h, respectively. When the model was pre-incubated with free OX26 to saturate the TfR on the BBB, the transport ratios of OX26-PL/pC27 and OX26/CTX-PL/pC27 decreased significantly, which displayed no difference with that of PL/pC27. There was no obvious alternation for PL/pC27 preconditioned with and without OX26. The results revealed OX26 modification on the PL/pDNA complexes played a critical role in the transport assay while CTX did not exert effect on the transport across BBB, and the increased transport ability of the PL modified with OX26 may be mediated by TfR. Our results were supported with the research that the TF-conjugated liposomes were able to increase the delivery of drug across the BBB mediated by TfR [40].

To evaluate the dual-targeting effects *in vitro*, the toxicity test of the lipoplexes on C6 glioma cells after crossing the BBB was conducted with the BBB/C6 co-culture model. As shown in Figure 4B, the viability of C6 cells were 89.9% for PL/pC27, 59.7% for OX26-PL/pC27, 46.0% for OX26/CTX-PL/pC27, 88.1% for PL/pC27 preconditioned with OX26, 79.0% for OX26-PL/pC27 preconditioned with OX26, 74.1% for OX26/CTX-PL/pC27 preconditioned with OX26, respectively. The OX26/CTX-PL/pC27 complexes exhibited enhanced cytotoxicity. Furthermore, after pre-incubation with OX26, the cytotoxicity of OX26/CTX-PL/pC27 was notably reduced and no differences were displayed, compared with that of PL/pC27, and OX26-PL/pC27 (p > 0.05). The results indicated evident dual-targeting effects of OX26/CTX-PL/pC27 due to the elevated endocytosis mediated by the specific binding CTX to MMP-2, following the increased transport across the BBB by the specific binding OX26 to TfR.

**Figure 5:**
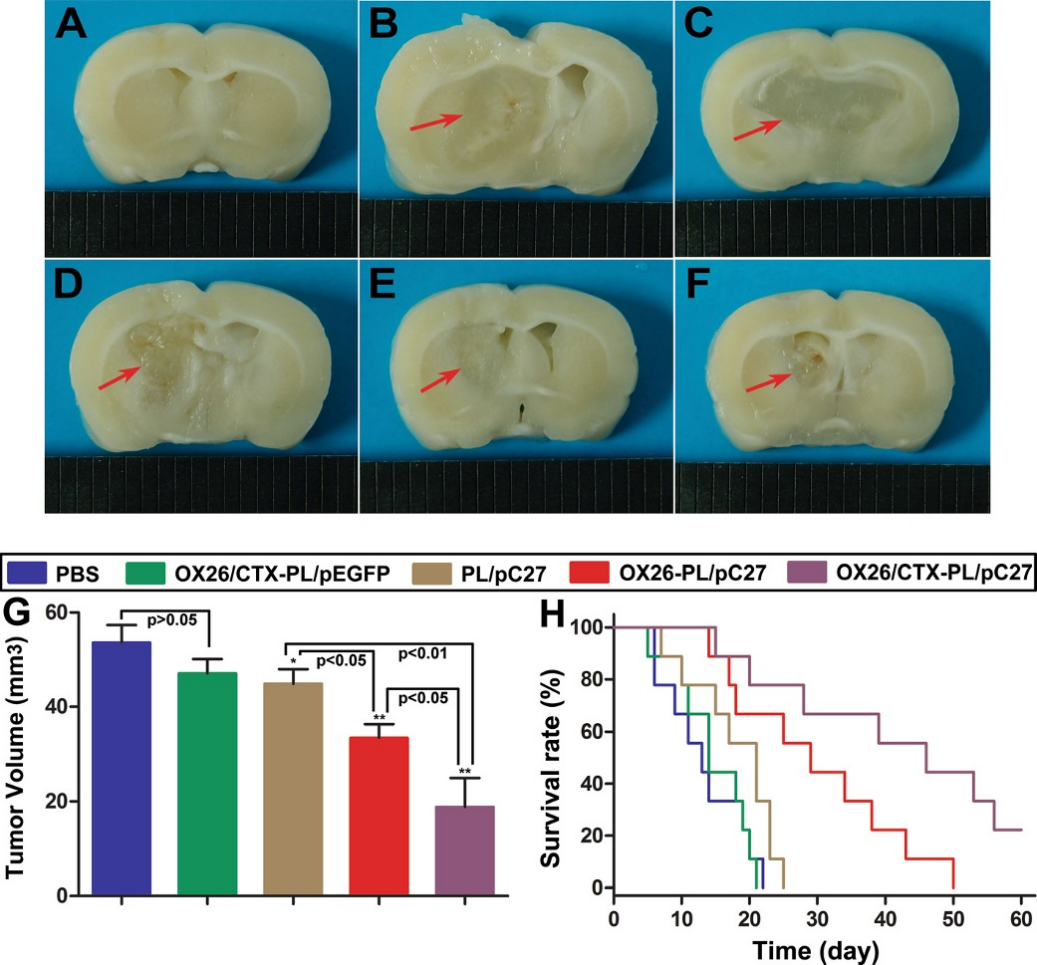
**Dual-targeting effects of OX26/CTX-PL/pC27 complex and survival monitoring** ***in vivo*** **. (A)** ~ **(F)**: the cross-sections of the rat brain among different treatment groups. **(A)** sham-operated group (without C6 cells or drug administration); **(B)** PBS control group; **(C)** OX26/CTX-PL/pEGFP control group; **(D)** PL/pC27 group; **(E)** OX26-PL/pC27 groups; **(F)** OX26/CTX-PL/pC27 group. The red arrows designated the tumor sites. **(G)** The tumor volumes of the C6 glioma bearing rats among different treatment groups. Data were presented as mean ± SD (n = 6). *p > 0.05, **p < 0.01, vs. OX26/CTX-PL/pEGFP. **(H)** The Kaplan-Meier survival curves of C6 glioma bearing rats treated with PBS, OX26/CTX-PL/pEGFP, PL/pC27, OX26-PL/pC27 and OX26/CTX-PL/pC27complexes after inoculation, respectively.

The rat C6 glioma models were employed in our study for further verifying the dual-targeting treatment effect of OX26/CTX-PL/pC27 *in vivo* (Figure 5A ~ F). The average tumor volumes on day 18 were 53.61 ± 3.71 mm^3^ for PBS group, 47.1 ± 3.02 mm^3^ for OX26/CTX-PL/pEGFP group, 44.87 ± 3.12 mm^3^ for PL/C27 group, 33.49 ± 2.83 mm^3^ for OX26-PL/pC27 group and 18.81 ± 6.15 mm^3^ for OX26/CTX-PL/pC27 group, respectively. As shown in Figure 5G, the OX26/CTX-PL/pC27 therapy significantly diminished the tumor size when compared with the controls (p < 0.01), PL/pC27 (p < 0.01) and OX26-PL/pC27 therapy (p < 0.05). On the contrary, OX26/CTX-PL/pEGFP did not exhibit inhibitory effects on the tumor, compared with the PBS control (p > 0.05). Treatment effects of different PL complexes were also reflected by the Kaplan-Meier survival curves (Figure 5H). The median survival time of rats treated with OX26/CTX-PL/pC27complexes (46 days) was significantly longer than that of rats treated with PBS (13 days, p = 0.000), OX26/CTX-PL/pEGFP (14 days, p = 0.000), PL/pC27 (21 days, p = 0.002) and OX26-PL/pC27 (29 days, p = 0.038), respectively. These results indicated that OX26-PL/pC27 was able to improve the treatment efficacy while the dual-targeting OX26/CTX-PL/pC27 led to the most significant tumor inhibition and the most significant improvement in the median survival time of tumor-bearing rats. These findings offered the robust evidence for the dual-targeting therapeutic effects.

**Figure 6:**
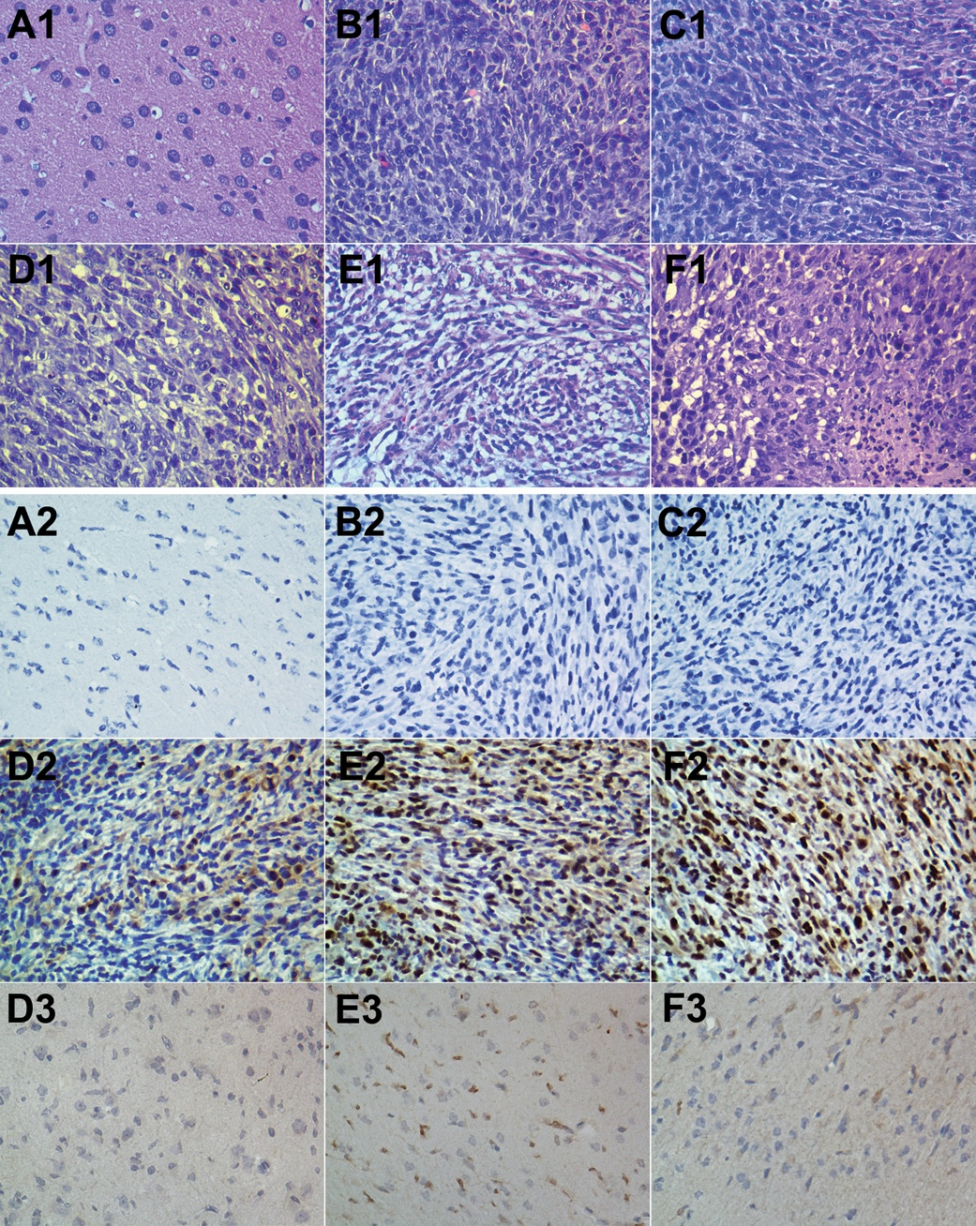
**HE staining (A**_**1**_ **~ F**_**1**_ **) and immunohistochemistry (A**_**2**_ **~ F**_**3**_ **) of brain tissues from the C6 glioma bearing rats (×400). A (A**_**1**_ **, A**_**2**_ **)**: Normal brain; **B (B**_**1**_ **, B**_**2**_ **)**: PBS-control; **C (C**_**1**_ **, C**_**2**_ **)**: OX26/CTX-PL/pEGFP group; **D (D**_**1**_ **, D**_**2**_ **, D**_**3**_ **)**: PL/pC27 group; **E (E**_**1**_ **, E**_**2**_ **, E**_**3**_ **)**: OX26-PL/pC27 group; **F (F**_**1**_ **, F**_**2**_ **, F**_**3**_ **)**: OX26/CTX-PL/pC27 group. **D**_**2**_, **E**_**2**_, **F**_**2**_ were from tumor cells; **D**_**3**_, **E**_**3**_, **F**_**3**_ were from normal cells adjacent to cancer.

To observe the histopathological changes and confirm the expression of the hTERTC27 protein, HE staining and IHC analysis were conducted. The hTERTC27 histochemistry showed that the gene was expressed only in the tumor site by EPR effects in the PL/pC27 group (Additional file 9: Figure S7B), and widely expressed throughout the brain in the OX26-PL/pC27 group (Additional file 9: Figure S7C). However, compared with that of the OX26-PL/pC27 group, the hTERTC27 protein mainly occurred in the tumor site and there was marked reduction in the expression out of the tumor site in the OX26/CTX-PL/pC27 group (Additional file 9: Figure S7D). As shown in Figure 6(A_1_ ~ F_1_), all glioma tissues were hypercellular with abundant cytoplasm, nuclear pleomorphism and partly with large nucleolus. In the OX26-PL/pC27 group, vacuolization was observed in most of the tumor cells and small, scattered necrosis happened sometimes (Figure 6E_1_). However, apoptotic bodies, nuclear fragments and massive necrosis occurred on the basis of the vacuolization in the OX26/CTX-PL/pC27 group (Figure 6F_1_). Further IHC study was performed to observe the expression of the hTERTC27 protein in the tumor cells and normal cells adjacent to cancer. The nuclei were stained blue and no hTERTC27 distributed in the tumor cells both in the PBS-control and OX26/CTX-PL/pEGFP groups (Figure 6B_2_, C_2_). In the PL/pC27, OX26-PL/pC27 and OX26/CTX-PL/pC27 groups, immunostaining of hTERTC27 protein was visualized throughout the cell, though primarily in the cell nuclei of glioma, and the scores were 1.33 ± 0.58, 7.33 ± 1.16 and 11.00 ± 1.73 in tumor cells while the scores were 0, 3.33 ± 1.15 and 8.00 ± 1.73 in normal cells, respectively (Figure 6D_2_ ~ F_3_). The staining score of tumor cells in the OX26/CTX-PL/pC27 group was significantly higher than that in PL/pC27 (p < 0.01) and OX26-PL/pC27groups (p = 0.034). On the contrary, the normal cells scored in the OX26/CTX-PL/pC27 group much lower than in the OX26-PL/pC27group (p < 0.01), which was consistent with the result of hTERTC27 gross distribution in rat brains. Immunohistochemical analysis further revealed the therapeutic effects derived from hTERTC27 expression in the tumor site.

In a word, compared with the existing studies, our dual-targeting delivery system was designed with the following features: (1) this system was of low toxicity and good biocompatibility; (2) the dual-targeting delivery increased therapeutic gene expression in the tumor site and improved the drug utilization; (3) intravenous administration avoided the damage caused by local injection and conformed to the development direction of nanocarriers; (4) the PEGylated liposomes, dual-targeting delivery, gene therapy and intravenous administration were fused together, and achieved a new breakthrough for the treatment of the glioma.

## Conclusions

The PEGylated liposomes modified with OX26 and CTX are able to significantly promote cell transfection, increase the transport of plasmid DNA across the BBB and afterwards target the brain glioma cells *in vitro* and *in vivo*, exhibit the most significant cytotoxicity *in vitro* and therapeutic efficacy in brain glioma-bearing rats. The ligand OX26 plays a critical role in transporting the lipoplexes across the BBB, and CTX acts as a major role in targeting brain glioma cells. Furthermore, OX26 also contributes to the glioma-targeting effect of the lipoplexes. The results would encourage further developments for non-invasive targeting therapy of brain gliomas by intravenous injection.

## Materials and methods

### Materials

Soybean phosphatidylcholine (S100-PC) was purchased from Lipoid GmbH (Ludwigshafen, Germany). DC-chol, 2-Iminothiolane hydrochloride (2-IT, Traut’s reagent), CTX and sulforhodamine B (SRB) were provided by Sigma-Aldrich (Saint Louis, MO, USA). Mal-PEG2000-DSPE was obtained from Avanti Polar Lipids (Alabaster, AL, USA). Anti-Transferrin Receptor antibody (OX-26) and anti-Telomerase reverse transcriptase antibody were purchased from Abcam (Cambridge, UK). Sephadex G-50 and Sepharose CL-4B were provided by Science and Technology Development Co., Ltd. (Beijing, China). Centriprep-10 concentrators were purchased from Millipore Amicon (Bedford, MA, USA). NanoOrange® Protein Quantitation Kit was purchased from Invitrogen (California, USA). YOYO-1 and LysoTracker Red were purchased from Molecular Probes (Eugene, OR, USA). pIRES2-EGFP (pEGFP) and pIRES2-EGFP/hTERTC27 (pC27) were purified using a E.Z.N.A.TM Fastfilter Plasmid Maxi Kit (Omega, Norcross, GA, USA). Cell Counting Kit-8 (CCK-8) was purchased from Dojindo Molecular Technologies (Tokyo, Japan). Lipofectamine™ 2000 Reagent (Lipo2000) was purchased from Invitrogen (California, USA). pGL3 Luciferase Reporter Vectors (pGL3-luc) and Luciferase Reporter Assay Kit were purchased from Promega (Madison, WI, USA).

### Cell culture

The C6, F98 glioma cells and human embryonic kidney 293 T (HEK 293 T) cells were obtained from ATCC (American Type Culture Collection, VA, USA) and were cultured in high glucose Dulbecco’s modified Eagle’s medium (DMEM, Gibco) supplemented with 10% fetal bovine serum (FBS, Gibco), 100 U/ml penicillin and 100 μg/ml streptomycin at 37°C in a humidified 5% CO2 incubator. Brain microvascular endothelial cells (BMVECs), were isolated from bovine brain and cultured in endothelial cell culture medium (DMEM, 20% FBS, 100 U/ml penicillin, 100 μg/ml streptomycin, 2 mmol/L L-glutamine, 100 μg/mL endothelial cell growth factor (ECGF), 20 μg/mL heparin, and 40 μU/mL insulin) [41].

### Animals

Male Wistar rats (250-280 g) were supplied by Laboratory Animal Center of Sun Yat-sen University (Guangzhou, China) and were housed in air-conditioned rooms (temperature: 21 ± 2°C; humidity: 55 ± 4% and light: 12 h light/dark cycle). All animal experiments were carried out with the approval of the Ethics Committee of Sun Yat-sen University.

### Preparation and characterization

#### Pegylated liposomes (PLs) synthesis and plasmid DNA encapsulation

PLs were prepared by reverse phase evaporation method as described previously [42, 43]. Briefly, 25.1 mg S100-PC, 19.9 mg DC-chol, 10.5 mg DSPE-PEG, 8.8 mg Mal-PEG2000-DSPE were dissolved in chloroform. The solution was then evaporated using rotary evaporation to form thin lipid film at room temperature. The dried lipid film was subsequently re-dissolved in chloroform and 0.5 mg plasmid of pEGFP or pC27 in 1 mL PBS was added. After vortex and ultrasonication at 10°C for 5 min, respectively, the solution was evaporated at 30°C to form a transparent and opalescence aqueous phase. The aqueous phase was extruded by turns through a 400 nm, 200 nm and 100 nm polycarbonate filter for 4 times, and the unmodified PL/pEGFP and PL/pC27 were formed. The encapsulation efficiency was determined as described previously [44].

#### Preparation of OX26 and/or CTX-conjugated liposome complexes

The OX26 and CTX were thiolated by using a 40:1 molar excess of 2-iminothiolane (Traut’s reagent) at room temperature for 1 hour, as described previously [6]. The thiolated OX26 and CTX (OX26-SH and CTX-SH) were incubated with PL/pEGFP and PL/pC27 (the weight ratio of peptide to phospholipid was 1:20) overnight at room temperature [45]. The targeting liposome complexes were separated using a Sepharose CL-4B column and eluted with 0.001 M PBS buffer (PH 7.4) from the reaction mixture [46]. The amount of uncoupled OX26 or CTX was determined according to the NanoOrange® Protein Quantitation Kit (Invitrogen Corporation, USA) [47]. The total and uncoupled amounts of OX26 or CTX were signed C_0_ and C_1_, respectively, coupling efficiency (CE) was calculated by the formula: CE = (1 − C_1_/C_0_) × 100%.

#### Particle size distribution, zeta potential and stability of liposome complexes

After the liposome complexes were dispersed in deionized water to make the solution with the final concentration of 0.25 mg/mL, the particle size distribution and zeta potential were determined by the light scattering method using a Zeta Potential/Particle Size analyzer (Zetasizer Nano ZS90, Malvern, UK) with a scattering angle of 90° at 25°C [48]. The morphology of the liposome complexes were observed using transmission electron microscopy (TEM) (JEM-2010, JEOL, Japan).

In order to determine the size stability of the liposome complexes, the samples (10 mg/ml) were kept in PBS (pH 7.4) (1:10, v/v) at 4°C for 1 week, and then the size change was determined by the analyzer above [49]. In addition, 100 μl samples (10 mg/ml) were added to 1 ml culture medium containing 10% FBS, and incubated at 37°C for 8 h. At 1 h, 4 h and 8 h, the particle size was measured to evaluate the variations in size [36].

### *In vitro*cytotoxicity assay of the modified and unmodified PL/pEGFP complexes

The cytotoxicity of the PL/pEGFP, OX26-PL/pEGFP and OX26/CTX-PL/pEGFP complexes was measured by CCK-8 assay. C6, HEK293T cells were seeded in a 96-well culture plates (Corning-Coaster, Tokyo, Japan) at 7000 cells per well in 100 μl DMEM medium for 24 h to achieve about 70% confluence. The medium was then replaced with 200 μl solution of different liposome complexes, which were loaded with a series of weights of pEGFP (0.1 μg, 0.2 μg, 0.4 μg, 0.8 μg, 1.2 μg, 1.6 μg), for 4 h. Then, cytotoxicity was performed using CCK-8 Kit. Cells without treatment were used as control. Absorbance was measured at 450 nm and corrected at 630 nm by dual wave length detection with TECAN Infinite M200 microplate reader (Tecan, Durham, USA). Cell viability of each group was expressed as a percentage relative to that of control.

### *In vitro*transfection experiment

C6, F98 and HEK293T cells were incubated in 24-well plates for 24 h to reach about 70% confluence. Before transfection, the medium was removed and the cells were washed 2 times with PBS. The cells were incubated with PL/pEGFP, OX26-PL/pEGFP and OX26/CTX-PL/pEGFP solutions containing 0.5 μg, 1 μg, 2 μg pEGFP in serum-free DMEM medium for 4 h at 37°C. After exchanging with complete medium, the cells were further incubated 48 h. The positive controls were treated with Lipo2000/pEGFP solutions containing the corresponding amounts of pEGFP while the negative one incubated with PBS only. The fluorescent images were observed and captured by the Eclipse TE2000-U fluorescence microscope (Nikon, Tokyo, Japan). For transfection efficiency analysis, the cells were trypsinized, washed and resuspended in 1.5 ml PBS. Transfection efficiency was evaluated by scoring the percentage of cells expressing green fluorescence protein (EGFP) using a FACS AriaTM System (Becton-Dickinson, San Joes, CA). The experiments were performed in triplicates and 15,000 cells were counted in each experiment.

### Luciferase reporter assay

Cells were transfected with the complexes containing 0.5 μg, 1 μg, 2 μg pGL3-luc in the 24-well plates in triplicate as described above. After 48 h of incubation, the cells were lysed by 100 μl lysis buffer for each well. The gene expression level in the lysates was evaluated using a luciferase reporter assay kit with a luminometer (Lumat LB9501 instrument, Bad Wildbach, Germany). The data were expressed as relative light units (RLU) per well of total cell protein.

### Intracellular expression and distribution of hTERTC27

The C6 glioma cells were seeded at a density of 1 × 10^4^ cells/well into 35 mm glass-bottom culture dishes (NEST, Wuxi, China) and cultured overnight. Then the cells were transfected with OX26/CTX-PL/pC27 solutions containing 4 μg pC27. C6 cells were washed three times with PBS, fixed with 4% paraformaldehyde at 0, 12, 24, 48 h, respectively. Then the cells were washed with PBS and incubated with Lyso-Tracker Red (1:10000, 30 min) and DAPI staining solution (5 min) to visualize the lysosomes and nuclei, respectively. The prepared cells were observed using laser scanning confocal microscope (Carl Zeiss LSM700, Jena, Germany).

### *In vitro*effects on C6 cells of the modified and unmodified PL/pC27 complexes

The C6 glioma cells were transfected with OX26/CTX-PL/pC27 complexes loading with a series of weights of pC27 (0.1 μg, 0.2 μg, 0.4 μg, 0.8 μg, 1.2 μg, 1.6 μg) in 96-well culture plates. After the cells were cultured in the complete DMEM culture medium for 48 h, cytotoxicity was performed using CCK-8 Kit. The cytotoxicity of PL/pC27, OX26-PL/pC27 and OX26/CTX-PL/pC27 containing 0.2 μg pC27 was also assessed by CCK-8 Kit. Each experiment was repeated for three times.

### Western blotting analysis

The C6 glioma cells were transfected with OX26/CTX-PL/pEGFP, PL/pC27, OX26-PL/pC27 and OX26/CTX-PL/pC27complexes containing 4 μg pDNA in 6-well plates. After the cells were incubated with complete culture medium for 48 h and total protein was extracted. Western blotting analysis was conducted as previously described [16]. Briefly, protein samples (20 μg) were separated using SDS-PAGE and transferred onto polyvinylidene difluoride (PVDF) membrane. The membrane was incubated at room temperature for 1 hour in a blocking buffer (5% low fat milk), and was then incubated with a rabbit primary antibody (1:1000 dilution) against the hTERTC27. Secondary antibody, horseradish peroxidase (HRP)-conjugated anti-rabbit IgG, was used to amplify the signal. The blots were developed using chemiluminescence system (New Life Science Products, Boston, MA, USA) and the results were photo-documented.

### *In Vitro*BBB model

The BBB model was established using BMVECs as described previously [50]. Briefly, 2% gelatin was pre-coated on 12-well cell culture inserts (polyethylene terephtalate membrane, 3 μm pore size, Millipore, Billerica, USA) for 30 min. Then BMVECs were seeded into the inserts at a density of 7.5 × 10^4^ cells/insert on day 1. The culture mediums were changed every other day. 4 h permeation assay was conducted and transendothelial electrical resistance (TEER) values of the BBB were measured with the TEER instrument (Word Precision Instruments, Sarasota, FL, USA) on day 5. Only the BBB models with no medium permeation in 4 h and the TEER value over 250 Ωcm^2^ were included for experiments.

### Transport across the BBB and competition assay

Lipoplexes, including PL/pC27, OX26-PL/pC27 and OX26/CTX-PL/pC27, in which pC27 was labeled by YOYO-1 [51], were added into the corresponding inserts at the plasmid concentration of 50 μg/mL. For the competition assay, excessive amount of OX26 (100 μg) was added into the inserts in advance for 30 min, and then cells were treated with PL/pC27, OX26-PL/pC27 and OX26/CTX-PL/pC27. A volume of 400 μl sample was taken from the acceptor compartments at 1, 2, 3, 4 h, and 400 μl fresh medium was added immediately after each sampling. The effects of all lipoplexes on BBB integrity were monitored by measuring TEER values during the experiment. The BBB transport ratios of pC27 were determined by RF-5301PC fluorospectrophotometry (Shimadzu, Japan) with the excitation and emission wavelength at 491 and 509 nm, respectively.

### Dual-targeting effects *in vitro*

A BMVECs/C6 cells co-culture model was established as previously reported [41]. The BBB model was established and then the inserts were transferred to another 12-well plate where C6 cells had been cultured for 1 day. Lipoplexes, including PL/pC27, OX26-PL/pC27 and OX26/CTX-PL/pC27, were added into the inserts of the BBB models at the plasmid concentration of 50 μg/mL for 4 h, respectively, and then inserts were removed. After another 48 h, the survival percentage of C6 glioma cells in the basolateral compartment was determined by SRB stainig assay.

### Dual-targeting effects and survival monitoring *in vivo*

Rat orthotopic glioma model was established as previously described [16]. Briefly, male Wistar rats (250–280 g) were anesthetized with 10% chloral hydrate (4 ml/kg) and fixed in a stereotactic apparatus (Huaibei Zhenghua Instruments Co., Anhui, China). C6 glioma cells (1 × 10^6^ cells suspended in 10 μl PBS) were stereotactically implanted into the right caudate nucleus (3 mm lateral and 1 mm anterior to the bregma, 5 mm of depth) with a Hamilton syringe (Shanghai Libao Instruments Co., Shanghai, China).

At day 3 after the implantation, the rats were divided into five groups (15 rats per group). Rats in blank control group were administered with PBS. Rats in the other 4 groups were treated with OX26/CTX-PL/pEGFP, PL/pC27, OX26-PL/pC27 and OX26/CTX-PL/pC27complexes via the tail vein at a dose of 40 μg pDNA per rat, respectively. Administration was made every 3 days with total 5 doses per rat. At day 18, six rats of each group were sacrificed for measuring the tumor size. The tumor volume was calculated by summing up the cross-sectional areas using the formula: Volume = *a*^2^ × *b* × π/6 (*a* and *b* represent the width and length of the tumor). The rest of rats were used for monitoring the survival time, which was analyzed by the Kaplan-Meier survival curve.

### Histopathology and immunohistochemistry (IHC) study

Rat brains were collected and stored in 4% paraformaldehyde. The sections were cut at 3 ~ 5 mm thick from paraffin-embedded brain tissue. After deparaffinization, sections were stained with haematoxylin and eosin (HE). IHC assay was performed using a standard two-step technique as demonstrated previously [52]. Briefly, after antigen retrieval was carried out by 4 minutes’ high pressure method in Tris/EDTA buffer solution (pH 9.0), the sections were incubated with 10% normal goat serum at room temperature for 10 min to reduce nonspecific reaction. Subsequently, the sections were incubated with anti-Telomerase reverse transcriptase antibody (1:100) overnight at 4°C. The goat anti-rabbit IgG antibody-HRP polymer (ZSGB-BIO, Beijing, China) was used as a secondary link to DAB chromogen. Finally, the sections were counterstained with Mayer’s hematoxylin. For the evaluation of hTERTC27 IHC staining, a semi-quantitative scoring method was used [53]. The staining intensity was scored as weak (1+), moderate (2+), and strong (3+). The number of positive staining cells were evaluated as follows: 0% (0), <10% (1), 10 ~ 50% (2), 51 ~ 80% (3); >80% (4). The staining index (scores 0 ~ 12) for each case was obtained by multiplying the values of the two parameters (percentage of the positive cells and predominant intensity).

### Statistics analysis

Data were presented as mean ± standard deviation (SD). One-way analysis of variance (ANOVA) was used to determine significance among groups following the Bonferroni’s post-test. p < 0.05 was considered to be significant.

## Electronic supplementary material

Additional file 1: Figure S1: The effect of the weight ratio of DC-chol and pEGFP (A) or pC27 (B) on the particle size and zeta potential of PL/pDNA complexes. (TIFF 1 MB)

Additional file 2: Table S1: The particle size, PDI and zeta potential of the PL/pDNA complexes (*n* = 3). (DOCX 14 KB)

Additional file 3: Figure S2: The TEM images of OX26/CTX-PL/pC27. Scale bar represents 100 nm. (TIFF 1 MB)

Additional file 4: Table S2: Encapsulation efficiency (EE) for the PL/pDNA complexes (*n* = 3). (DOCX 13 KB)

Additional file 5: Figure S3: The cell viability of the HEK293T and C6 cells after incubation with PL/pEGFP, OX26-PL/pEGFP and OX26/CTX-PL/pEGFP complexes at 0.1, 0.2, 0.4, 0.8, 1.2, 1.6 μg of plasmid weights, respectively. (A) HEK293T cells, (B) C6 glioma cells. (TIFF 810 KB)

Additional file 6: Figure S4: Fluorescent microscopy images of HEK293T cells, C6 and F98 gioma cells after transfection of Lipo2000/pEGFP, PL/pEGFP, OX26-PL/pEGFP and OX26/CTX-PL/pEGFP complexes containing 1 μg pIRES2-EGFP. (TIFF 8 MB)

Additional file 7: Figure S5: Transfection efficiency of PL/pGL3-luc, OX26-PL/pGL3-luc and OX26/CTX-PL/pGL3-luc complexes containing 0.5 μg, 1 μg, 2 μg pGL3-luc in (A) HEK293T, (B) C6 glioma cells, and (C) F98 glioma cells. The RLU value is given on the Y-axis and data are expressed as mean ± SD (n = 3). (TIFF 2 MB)

Additional file 8: Figure S6: Confocal images of C6 cells after incubation with OX26/CTX-PL/pC27 for 0 h, 12 h, 24 h and 48 h. Blue (DAPI), Red (Lyso-Tracker Red) and Green (EGFP) represent nuclei, lysosomes and hTERTC27 expression, respectively. (TIFF 4 MB)

Additional file 9: Figure S7: hTERTC27 distribution in rat brains removed from (A) PBS control group; (B) PL/pC27 group; (C) OX26-PL/pC27 group; (D) OX26/CTX-PL/pC27 group. (TIFF 12 MB)

## Abbreviations

ATCC: American Type Culture Collection
BBB: Blood–brain barrier
BMVECs: Brain microvascular endothelial cells
CCK-8: Cell counting kit-8
CTX: Chlorotoxin
EGFP: Enhanced green fluorescence protein
EPR: Enhanced permeability and retention
GBM: Glioblastoma multiforme
HE: Haematoxylin and eosin
HEK 293T: Human embryonic kidney 293T
hTERT: Human telomerase reverse transcriptase
hTERTC27: 27 kDa C-terminal polypeptide of hTERT
IES: Interendothelial cell slit
IHC: Immunohistochemistry
2-IT: 2-Iminothiolane hydrochloride (Traut’s reagent)
Lipo2000: Lipofectamine™ 2000 Reagent
MMP-2: Matrix metalloproteinase-2
MPS: Mononuclear phagocyte system
OX-26: Anti-transferrin receptor antibody
OX26/CTX-PL/pC27: pC27-loaded PEGylated liposomes modified with OX26 and CTX
OX26/CTX-PL/pEGFP: pEGFP-loaded PEGylated liposomes modified with OX26 and CTX
pC27: Plasmid IRES2-EGFP/hTERTC27
pEGFP: Plasmid IRES2-EGFP
PDI: Polydispersity index
PEG: Polyethyleneglycol
pGL3-luc: pGL3 Luciferase Reporter Vectors
PLs: PEGylated liposomes
S100-PC: Soybean phosphatidylcholine
TEER: Transendothelial electrical resistance
TEM: Transmission electron microscope
Tf: Transferrin
TfR: Transferrin receptor.
