# Supplementary figures and images for: OX26/CTX-conjugated PEGylated liposome as a dual-targeting gene delivery system for brain glioma
Source: Mol Cancer. 2014 Aug 13;13:191. doi: 10.1186/1476-4598-13-191 (PMC4137094; doi:10.1186/1476-4598-13-191)

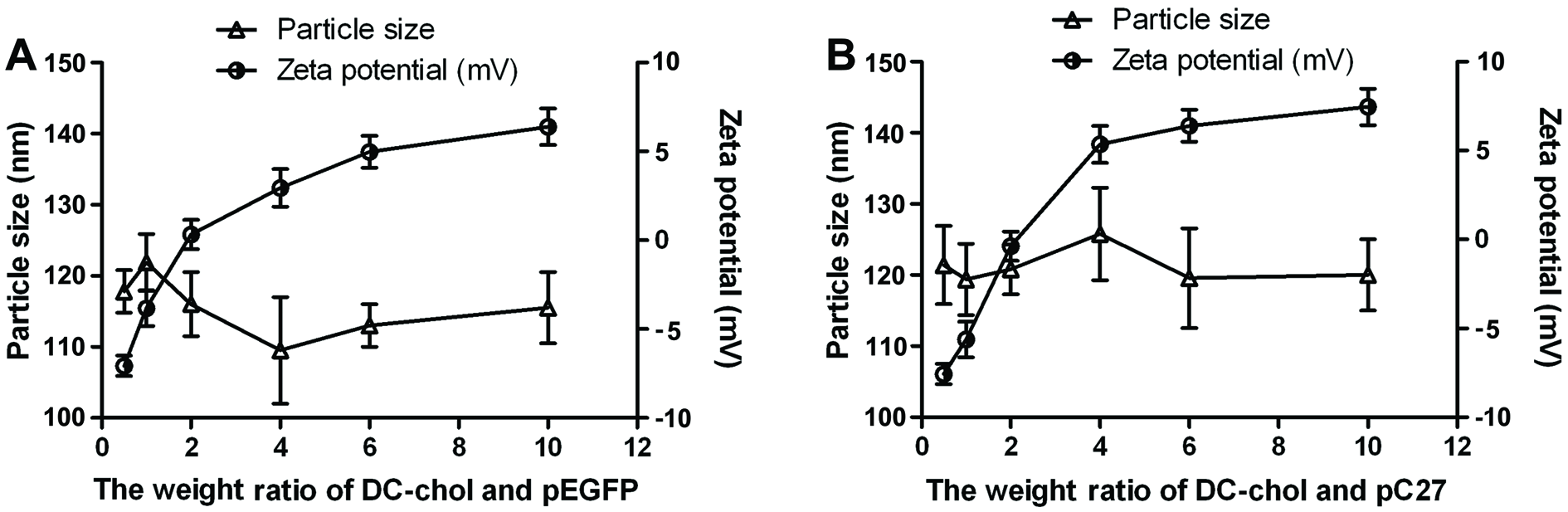

Supplement: Supplementary file 1 — Additional file 1: Figure S1: The effect of the weight ratio of DC-chol and pEGFP (A) or pC27 (B) on the particle size and zeta potential of PL/pDNA complexes. (TIFF 1 MB) [file 12943_2014_1390_MOESM1_ESM.tiff]

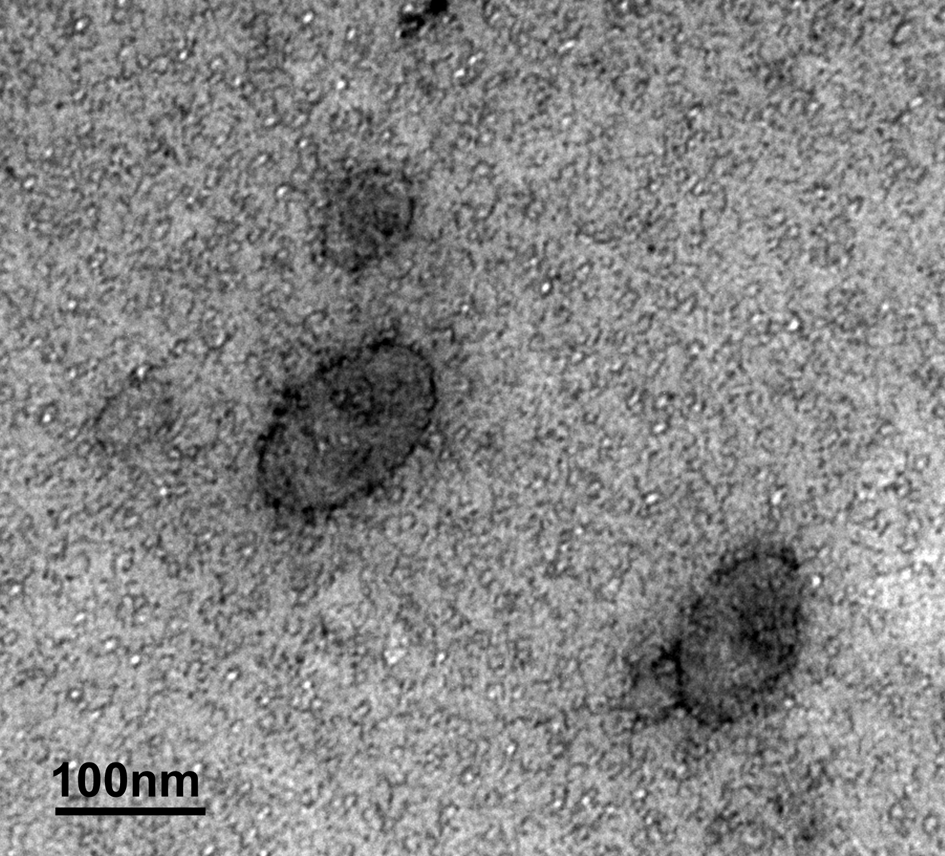

Supplement: Supplementary file 3 — Additional file 3: Figure S2: The TEM images of OX26/CTX-PL/pC27. Scale bar represents 100 nm. (TIFF 1 MB) [file 12943_2014_1390_MOESM3_ESM.tiff]

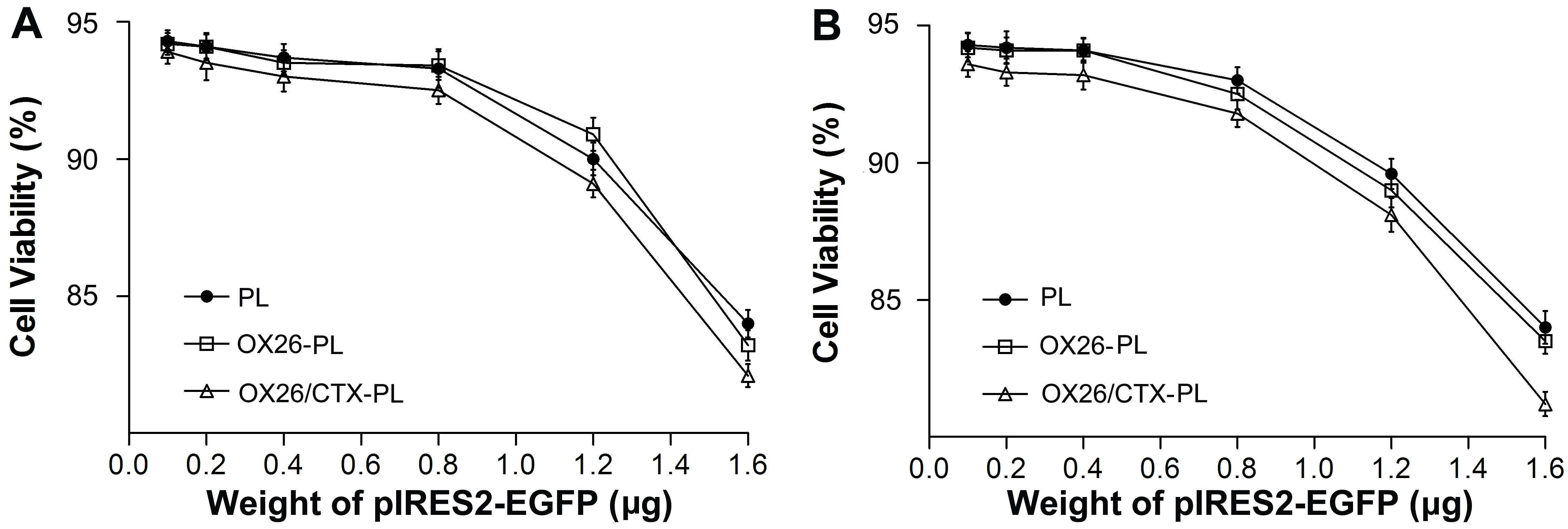

Supplement: Supplementary file 5 — Additional file 5: Figure S3: The cell viability of the HEK293T and C6 cells after incubation with PL/pEGFP, OX26-PL/pEGFP and OX26/CTX-PL/pEGFP complexes at 0.1, 0.2, 0.4, 0.8, 1.2, 1.6 μg of plasmid weights, respectively. (A) HEK293T cells, (B) C6 glioma cells. (TIFF 810 KB) [file 12943_2014_1390_MOESM5_ESM.tiff]

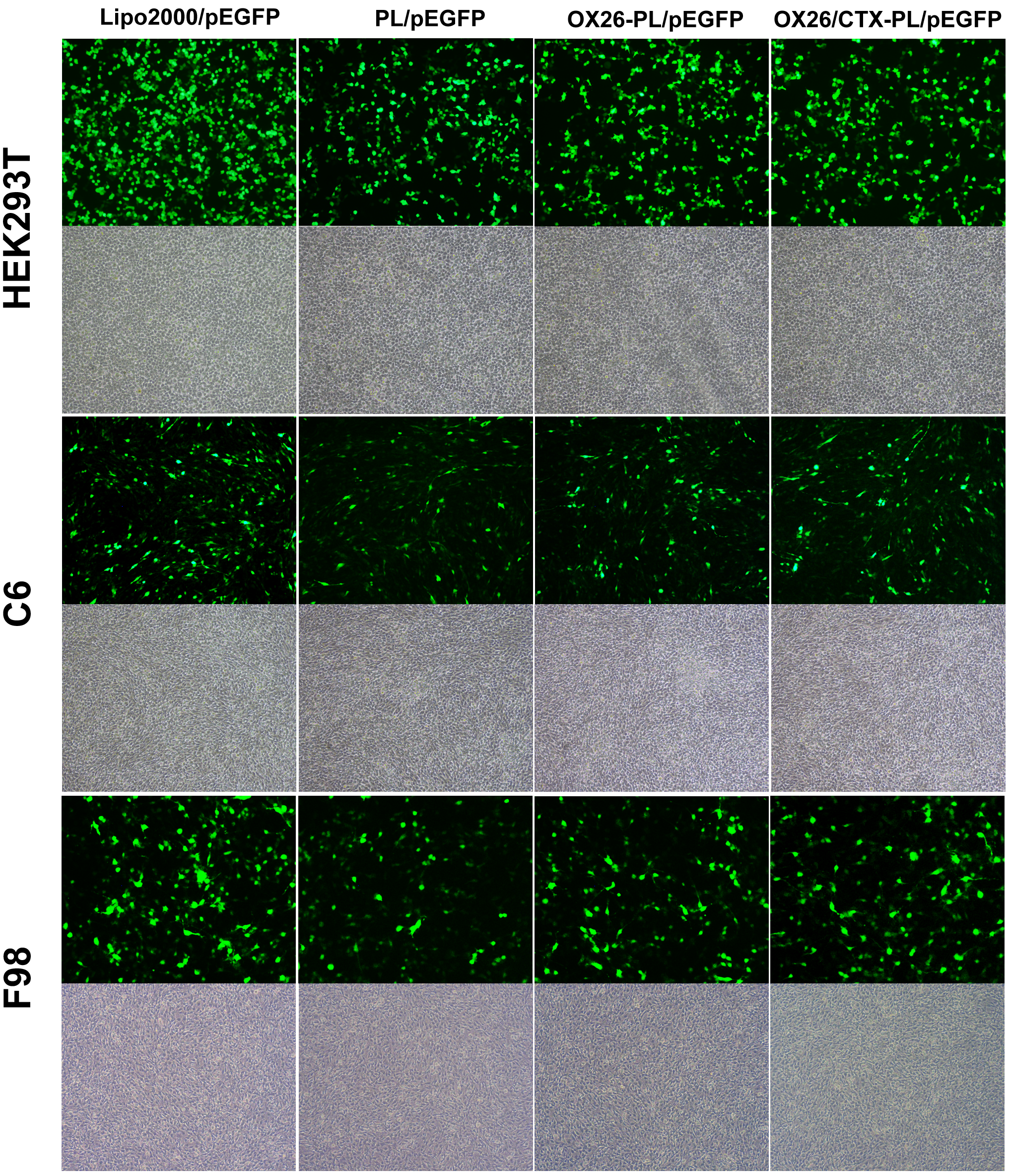

Supplement: Supplementary file 6 — Additional file 6: Figure S4: Fluorescent microscopy images of HEK293T cells, C6 and F98 gioma cells after transfection of Lipo2000/pEGFP, PL/pEGFP, OX26-PL/pEGFP and OX26/CTX-PL/pEGFP complexes containing 1 μg pIRES2-EGFP. (TIFF 8 MB) [file 12943_2014_1390_MOESM6_ESM.tiff]

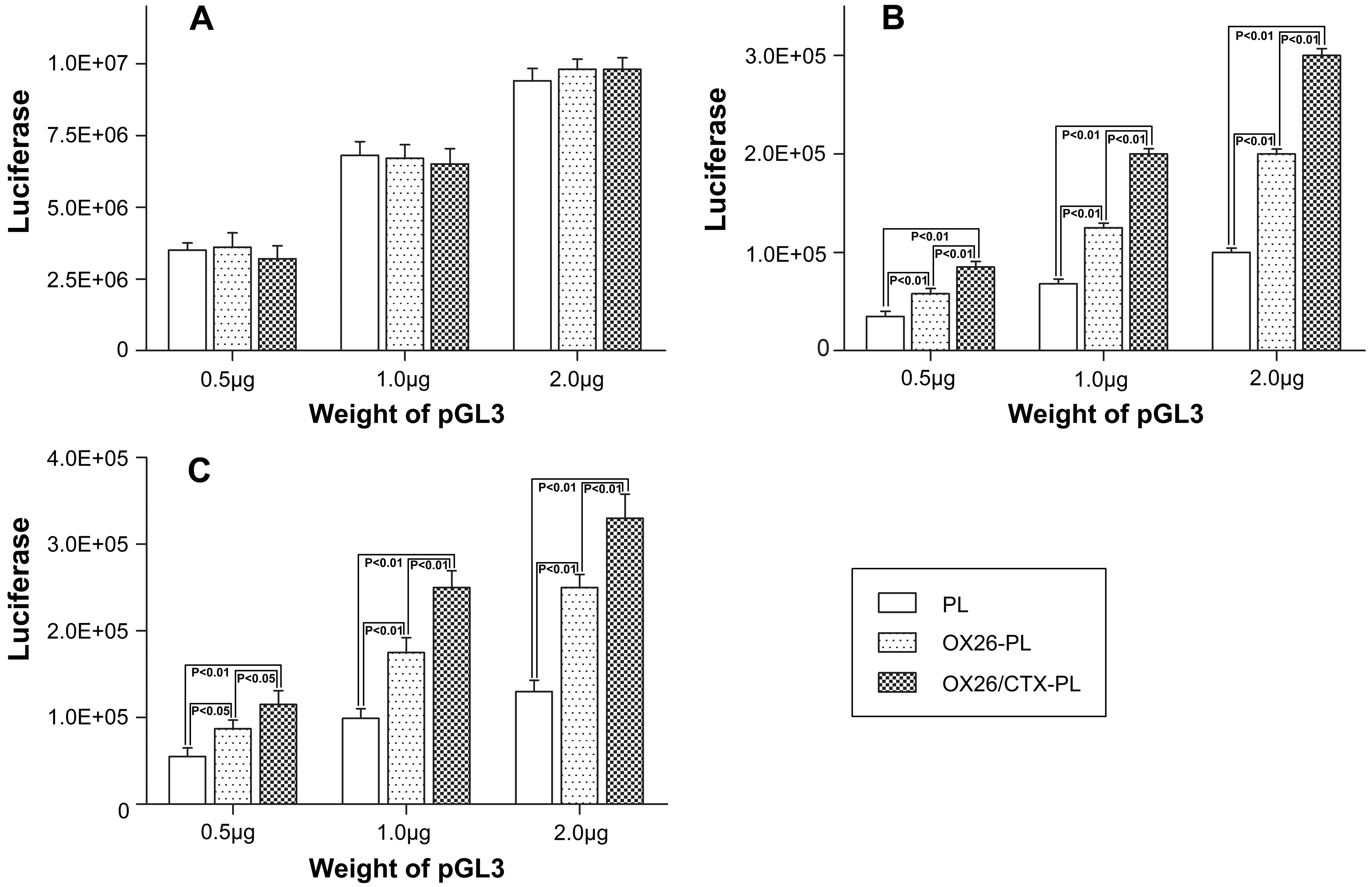

Supplement: Supplementary file 7 — Additional file 7: Figure S5: Transfection efficiency of PL/pGL3-luc, OX26-PL/pGL3-luc and OX26/CTX-PL/pGL3-luc complexes containing 0.5 μg, 1 μg, 2 μg pGL3-luc in (A) HEK293T, (B) C6 glioma cells, and (C) F98 glioma cells. The RLU value is given on the Y-axis and data are expressed as mean ± SD (n = 3). (TIFF 2 MB) [file 12943_2014_1390_MOESM7_ESM.tiff]

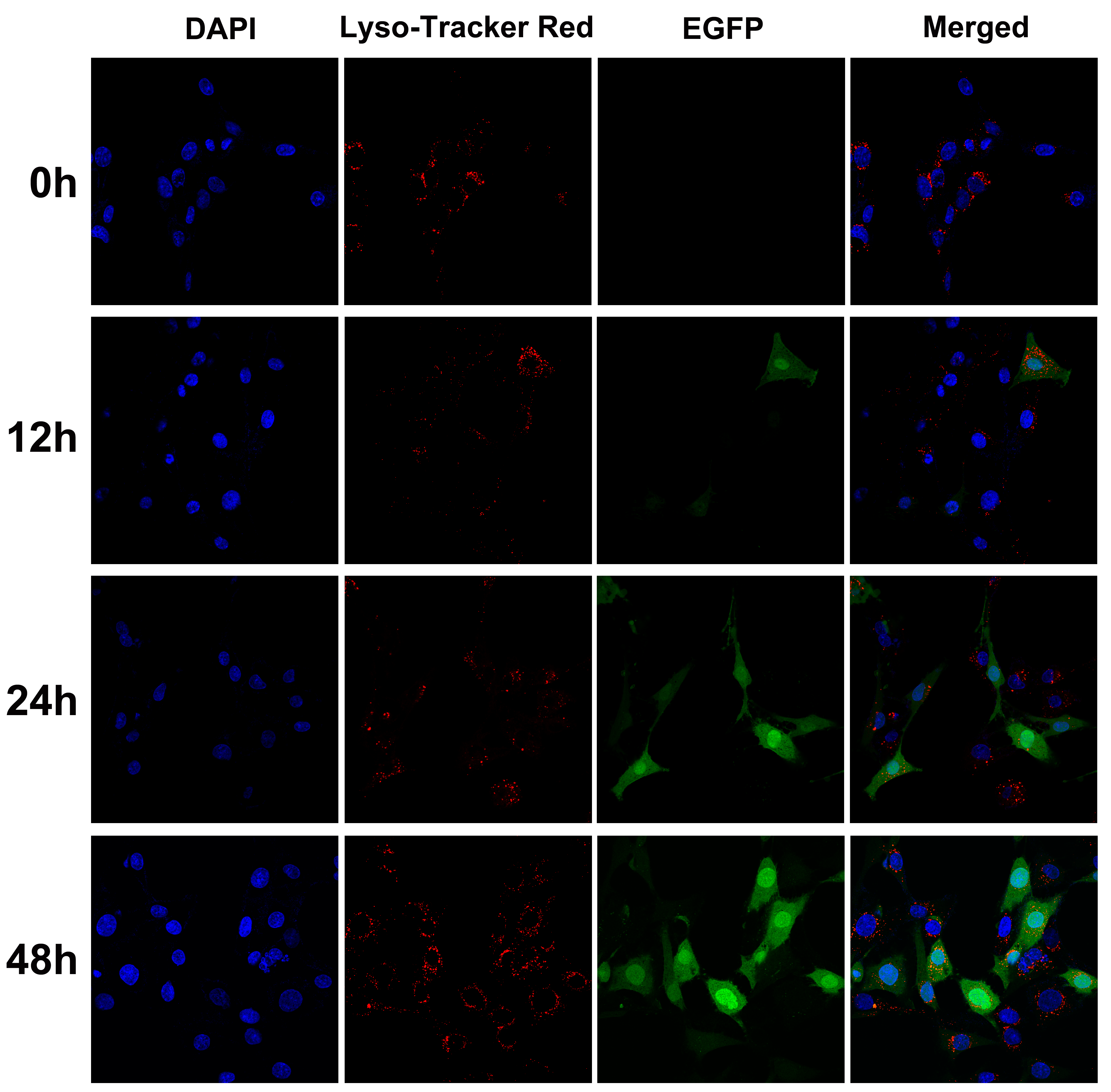

Supplement: Supplementary file 8 — Additional file 8: Figure S6: Confocal images of C6 cells after incubation with OX26/CTX-PL/pC27 for 0 h, 12 h, 24 h and 48 h. Blue (DAPI), Red (Lyso-Tracker Red) and Green (EGFP) represent nuclei, lysosomes and hTERTC27 expression, respectively. (TIFF 4 MB) [file 12943_2014_1390_MOESM8_ESM.tiff]

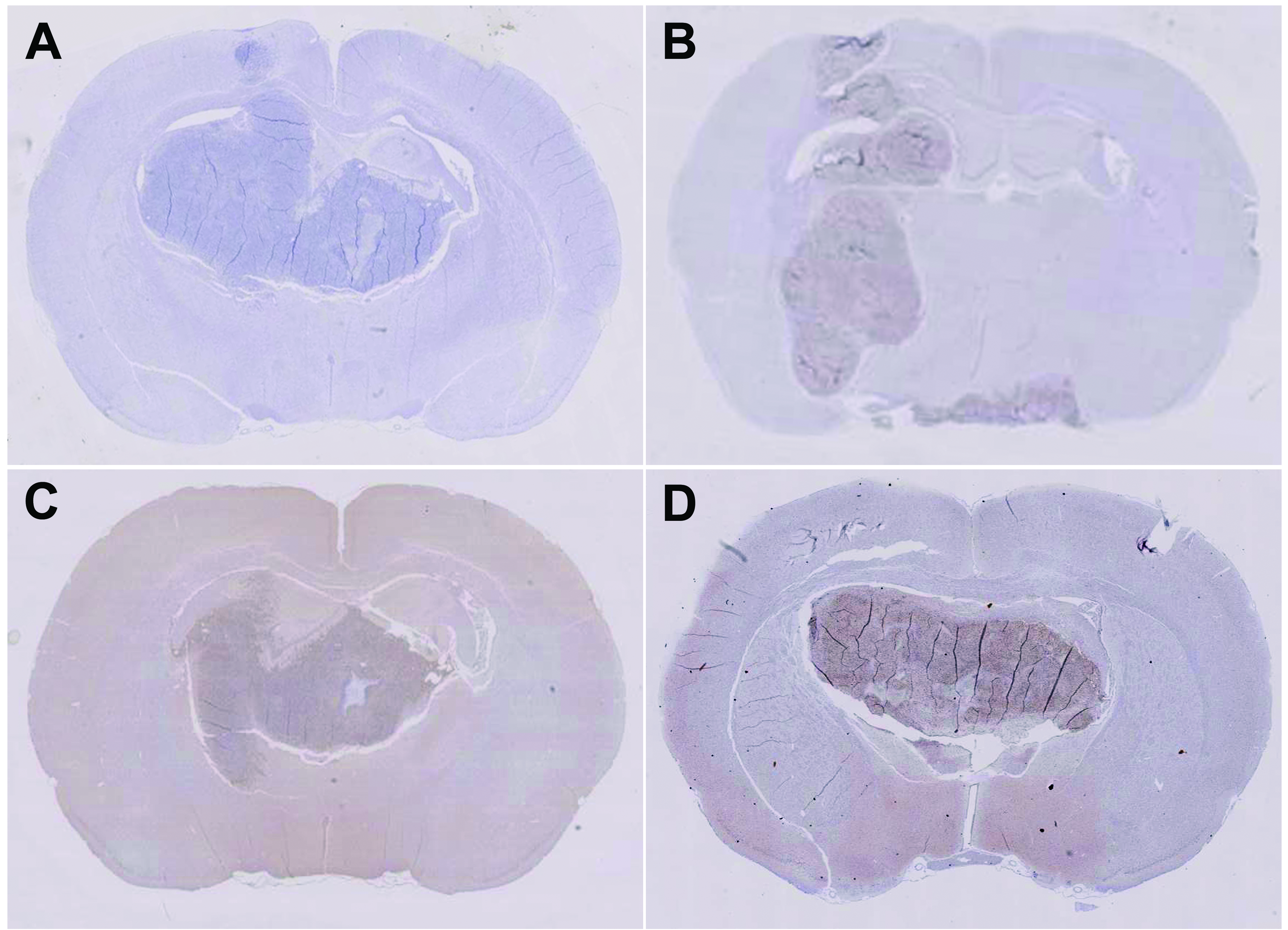

Supplement: Supplementary file 9 — Additional file 9: Figure S7: hTERTC27 distribution in rat brains removed from (A) PBS control group; (B) PL/pC27 group; (C) OX26-PL/pC27 group; (D) OX26/CTX-PL/pC27 group. (TIFF 12 MB) [file 12943_2014_1390_MOESM9_ESM.tiff]
