# Supplementary material for: OX26/CTX-conjugated PEGylated liposome as a dual-targeting gene delivery system for brain glioma
Source: Mol Cancer. 2014 Aug 13;13:191. doi: 10.1186/1476-4598-13-191 (PMC4137094; doi:10.1186/1476-4598-13-191)
Supplement: Supplementary file 2 — Additional file 2: Table S1: The particle size, PDI and zeta potential of the PL/pDNA complexes (n = 3). (DOCX 14 KB) [file 12943_2014_1390_MOESM2_ESM.docx]

**Table S1 The particle size, PDI and zeta potential of** **the PL/pDNA complexes (*n*=3)**

| Formulations | Particle size (nm) | PDI | Zeta potential (mV) |
| --- | --- | --- | --- |
| PL/pEGFP | 113.3±8.3 | 0.173±0.013 | 5.2±1.1 |
| OX26-PL/pEGFP | 121.8±9.9* | 0.175±0.021* | 6.2±1.4* |
| OX26/CTX-PL/pEGFP | 108.6±10.3* | 0.195±0.031* | 7.6±1.3* |
| PL/pC27 | 131.5±6.6 | 0.182±0.028 | 11.2±1.9 |
| OX26-PL/pC27 | 122.0±11.1* | 0.211±0.031* | 12.2±2.0* |
| OX26/CTX-PL/pC27 | 124.3±9.9* | 0.201±0.026* | 12.8±2.3* |

* p>0.05 versus pegylated liposomes with the same pDNA
