# Supplementary material for: OX26/CTX-conjugated PEGylated liposome as a dual-targeting gene delivery system for brain glioma
Source: Mol Cancer. 2014 Aug 13;13:191. doi: 10.1186/1476-4598-13-191 (PMC4137094; doi:10.1186/1476-4598-13-191)
Supplement: Supplementary file 4 — Additional file 4: Table S2: Encapsulation efficiency (EE) for the PL/pDNA complexes (n = 3). (DOCX 13 KB) [file 12943_2014_1390_MOESM4_ESM.docx]

**Table S2 Encapsulation efficiency (EE) for** **the PL/pDNA complexes (*n*=3)**

| Formulations | EE（%） |
| --- | --- |
| PL/pEGFP | 92.3±3.8 |
| OX26-PL/pEGFP | 91.3±4.5 |
| OX26/CTX-PL/pEGFP | 90.0±5.1 |
| PL/pC27 | 88.4±4.8 |
| OX26-PL/pC27 | 90.2±2.5 |
| OX26/CTX-PL/pC27 | 85.8±6.2 |
